# The Social Safeness and Pleasure Scale (SSPS): a psychometric evaluation of the Swedish version in a non-clinical sample and two clinical samples with eating disorders or borderline personality disorder

**DOI:** 10.1186/s40359-022-01020-2

**Published:** 2022-12-16

**Authors:** Martina Isaksson, Maria Holmbom Goh, Mia Ramklint, Martina Wolf-Arehult

**Affiliations:** 1 Department of Medical Sciences, Psychiatry, Uppsala University, Entrance 10, Floor 3B, 751 85 Uppsala, Sweden; 2 Department of Clinical Neuroscience, Centre for Psychiatry Research, Karolinska Institutet and Stockholm Health Care Services, Region Stockholm, Stockholm, Sweden

**Keywords:** Social Safeness and Pleasure Scale, Psychometric properties, Factor structure, Reliability, Validity, Eating disorders, Borderline personality disorder

## Abstract

**Background:**

Social safeness and pleasure refer to the extent to which people experience their world as safe, warm, and soothing. Difficulties in achieving social safeness have been identified as a transdiagnostic vulnerability factor for developing and maintaining psychopathology and for feeling less contentment and self-compassion. The study aim was to evaluate the psychometric properties of the Swedish version of the Social Safeness and Pleasure Scale (SSPS).

**Methods:**

The SSPS was evaluated in a non-clinical sample of 407 participants. The internal consistency and test–retest reliability of the SSPS were explored and a confirmatory factor analysis was performed. Convergent validity was studied based on the assumption of negative correlations with the personality traits detachment and mistrust, derived from the Swedish Universities Scale of Personality. Divergent validity was studied based on the assumption of no or small correlations with impulsiveness and adventure-seeking—personality traits not assumed to be related to social safeness. Validity was also investigated by comparing the SSPS results in the non-clinical sample with those in two clinical groups of patients diagnosed with either borderline personality disorder (BPD; n = 58) or eating disorders (n = 103), recruited from two psychiatric outpatient clinics.

**Results:**

Confirmatory factor analysis confirmed a one-factor structure. Cronbach’s alpha was 0.95 and test–retest reliability was 0.92. Validity was supported by moderate to strong negative correlations between the SSPS and the detachment and mistrust scales and no or small correlations with the impulsiveness and adventure-seeking scales in a personality questionnaire. Finally, we found significantly lower mean values on the SSPS in the clinical groups compared with the non-clinical group, with the lowest mean in the BPD sample.

**Conclusions:**

The results showed good to excellent psychometric properties for the Swedish version of the SSPS, supporting its use in both clinical practice and research. Future research could use the SSPS when evaluating interventions aimed at improving the ability to develop social safeness, such as compassion-focused therapy or radically open dialectical behavior therapy, interventions that may be particularly important in BPD patients.

## Background

Social safeness and pleasure refer to the extent to which people experience their world as safe, warm, and soothing. Even though these factors have been suggested to be important for mental health, the focus in both research and clinical psychiatric practice has thus far been more on negative than positive emotions or affects [1, 2].

From an evolutionary perspective, different types of affects have evolved for different reasons. At least three basic life tasks have been described in animals: (1) to detect, avoid, and protect themselves from threats, (2) to acquire, control, and maintain resources for survival and reproduction, and (3) to regulate affect and motivation during times of affiliation and goal satisfaction [3–5]. These tasks are thought to produce distinct types of affects: the first generating negative affect (NA) deriving from the threat and defense system, and the latter two promoting different types of positive affect (PA) [4–7]. An initial distinction between two different types of PA was made by Depue and Morreone-Strupinsky [8], describing a dopamine-linked PA associated with drive and arousal and a second PA linked to endogenous opiates and oxytocin and associated with contentment and feelings of well-being. Looking further into different types of PA, three distinct factors have been identified: feeling energized and excited, feeling relaxed and calm, and feeling safe and content [6]. Social PA (i.e., feeling safe and content among others) and non-social PA (i.e., feeling relaxed and calm in non-social contexts) seem to operate differently. To be able to talk about and assess only the aspect of social PA, the construct of social safeness has been suggested. This refers to the extent to which people experience their world as safe, warm, and soothing, and relates to feelings such as belonging, acceptance, and warmth from others [9]. The Social Safeness and Pleasure Scale (SSPS) is an 11-item self-rating instrument developed to assess social safeness on a 5-point Likert scale. It has been widely used and translated into several languages with good to excellent internal consistency, with Cronbach’s alpha ranging from 0.82 to 0.94 [6, 10, 11].

The SSPS was originally validated in undergraduate psychology students and patients with bipolar disorder [6]. An exploratory factor analysis in the sample consisting of the 202 undergraduate psychology students, 36 of which were male, indicated a one-factor structure. Further, results showed that non-social PA has few correlations with other PA, whereas PA deriving from social relationships (social safeness) was significantly related to other types of PA. In particular, robust correlations were found between social safeness and feelings of contentment and joy in both students and patients. In contrast, social safeness was not linked to vitality or excitement in either group. Results also showed that patients with high levels of social safeness reported smaller fluctuations in mood than patients with lower levels [6]. Social safeness has shown strong negative correlations with depression, anxiety, stress, self-criticism, and insecure attachment [9].

Further, difficulties in achieving social safeness and pleasure has been suggested as a transdiagnostic vulnerability factor for different psychological problems [2, 12]. There is also evidence that therapeutic interventions targeting social safeness and pleasure can be beneficial [13]. More specifically, social safeness has been suggested to be a kind of buffer against problems with mental health [6], as it affects the capacity for adaptive coping to the struggles of life and psychopathologies. For example, it may be easier to show compassion towards oneself and others when experiencing social safeness, and social safeness is believed to function as a shame antidote [13, 14]. Social safeness might also be a key mechanism through which recollections of parental warmth relate to a person’s capacity to develop self-compassion and receive compassion, whereas recollections of a lack of adequate warmth in a person’s early environment might lead to an underdeveloped soothing system [15].

Several studies have investigated social safeness and disordered eating in women. For example, in a non-clinical (NC) population of Portuguese women [16], findings showed that higher levels of social safeness were associated to a healthier attitude towards their own body and more flexible eating rules, which seemed to explain lower levels of disordered eating. Moreover, an interesting comparison of patients with different subtypes of eating disorders (ED) has been performed, analyzing changes in ED pathology during a 12-week treatment period [14]. Results revealed the slowest improvements in social safeness, self-compassion, received social support, and shame among patients with anorexia nervosa (AN). In particular, the slowest change in social safeness was reported for AN, binge-purge subtype [14].

Research has also suggested that low social safeness uniquely predicts borderline personality traits based on the Structured Clinical Interview for DSM-IV Axis II Personality Disorders (SCID-II) Self-Report Questionnaire [7]. This may not come as a surprise, since social safeness has also shown high negative correlations with self-criticism [9], and various forms and functions of self-criticism are known to be linked to self-harm [17]. In addition, the unstable relationships that are characteristic for patients with borderline personality disorder (BPD) [18] could be related to low social safeness. The causality of this relation is however speculative. Unstable relationships may increase the risk of developing feelings of low social safeness since social safeness seems to be affected by whether the individual has received social support. The opposite could however also be the case, i.e., lack of social safeness may contribute to unstable relationships.

In summary, social safeness represents a PA that operates differently from non-social PA, and can serve as protection against and mitigation of threat-driven NA and behaviors. It may therefore be useful to further investigate social safeness to improve the understanding of treatment effects and failures in clinical research. This is true in particular for EDs and BPD. The aim of the present study was to examine the psychometric properties of the Swedish version of the SSPS with both a NC sample and two clinical samples. The study has the potential to replicate previous findings of the SSPS and to extend knowledge of social safeness and pleasure in two additional clinical groups, i.e., the ED group and the BPD group. We hypothesized 1) that the scale would be comprised by one single factor, 2) that the scale would show adequate internal consistency and be stable over time, 3) that the scale would show adequate convergent and divergent validity when contrasted to scales measuring related and distinct constructs respectively, and 4) that the scale would show satisfactory construct validity, when comparing results between a NC sample and two clinical samples.

## Methods

### Procedures and participants

A NC control group was recruited through convenience sampling from January to December 2019 via advertisements in social media, on the homepage of the Department of Neuroscience in Uppsala, and on a website for people interested in participating in scientific research. An inclusion criterion was being at least 18 years old. Participants were offered two movie tickets for their participation. There were 467 participants, of whom 407 filled out the SSPS, constituting the NC sample. Two weeks after completing the first questionnaires, a group of NC participants received a new email with a request to fill out the questionnaires a second time. This was done by 49 participants, making it possible to calculate test–retest reliability. Demographic characteristics are presented in Table 1.

**Table 1:** Demographic characteristics of the participants

	NC sample (n = 407)	ED sample (n = 103)	BPD sample (n = 58)
Mean age, years (SD)	30.4 (10.7)	25.1 (6.4)	26.4 (7.0)
Age range, years	18–77	18–48	18–59
*Gender*^*a*^			
Women	303 (74.4%)	96 (93.2%)	53 (91.4%)
Men	103 (25.3%)	6 (5.8%)	5 (8.6%)
*Marital status*			
Single	175 (43.1%)	55 (54.5%)	24 (42.1%)
Married or in relationship	228 (56.2%)	43 (42.6%)	42 (56.1%)
Other (e.g., living with parents)	3 (0.7%)	3 (3.0%)	1 (1.8%)
*Highest level of education*			
Elementary school	11 (2.7%)	16 (15.5%)	19 (33.3%)
High school	119 (29.2%)	51 (49.5%)	30 (52.6%)
Higher education	277 (68.1%)	36 (35.0%)	8 (14.0%)
*Occupation*			
Paid work	176 (43.2%)	36 (35.3%)	17 (30.4%)
Student or internship	186 (45.7%)	47 (46.1%)	11 (19.6%)
Unemployed	26 (6.4%)	1 (1.0%)	8 (14.3%)
Sick leave (including disability pension)	17 (4.2%)	17 (16.7%)	18 (32.1%)
Other (e.g., parental leave)	1 (0.5%)	1 (1.0%)	2 (3.6%)

*NC* Non-clinical, *ED* Eating disorder, *BPD* Borderline personality disorder

^a^ Two participants did not report gender (one NC, one ED), ^b^ Four participants did not report marital status (one NC, two ED, one BPD), ^c^ One BPD participant did not report level of education, ^e^ Three participants did not report occupation (one ED, two BPD)

The clinical groups were recruited from 2014 to 2019 at the ED clinic and the BPD clinic at Uppsala University Hospital, Sweden. Inclusion criteria were (1) minimum age 18 years, and (2) being diagnosed with AN, bulimia nervosa (BN), other specified feeding or eating disorder, unspecified eating disorder, or BPD. Excluded were patients not able to fill out self-report questionnaires independently due to illness, e.g., having a need for immediate inpatient treatment, and patients with insufficient cognitive ability or limited ability to understand Swedish. Those willing to participate signed an informed consent form and were asked to fill out the pen-and-paper questionnaires at the clinic or at home. Patients received no compensation for participating. The clinical groups consisted of 103 patients with ED and 58 patients with BPD. Demographic characteristics are presented in Table 1.

### Instruments

#### The Social Safeness and Pleasure Scale (SSPS)

The SSPS is an 11-item self-rating instrument that assesses the extent to which people experience their world as safe, warm, and soothing, and how connected they feel to others. Items such as “I feel secure and wanted” are included and participants indicate their answers on a 5-point Likert scale, ranging from 1 (almost never) to 5 (almost all the time). Scores are added together to produce a total score in the range 11–55, with higher scores representing higher perceived social safeness and connectedness to others. The original English version of the SSPS was found to consist of one factor, and is considered highly reliable, with an excellent Cronbach’s alpha of 0.91 [6]. A professional translator performed a translation from English to Swedish, while another authorized translator conducted a translation back to English. The first author and the last author supervised the process and agreed on the final version in collaboration with Gilbert’s research group.

#### The Swedish Universities Scales of Personality (SSP)

The Swedish Universities Scales of Personality (SSP) is a 91-item self-report personality questionnaire measuring personality traits based on biological theories [19]. It was developed in part to create a test battery from which single scales could be extracted for particular research purposes and consists of 13 subscales: somatic trait anxiety, psychic trait anxiety, stress susceptibility, lack of assertiveness, impulsiveness, adventure-seeking, detachment, social desirability, embitterment, trait irritability, mistrust, verbal trait aggression, and physical trait aggression. The scale yields a three-factor solution consisting of factors reflecting neuroticism, aggressiveness, and a broad extraversion factor which can be subdivided into an impulsiveness/adventure-seeking factor and a detachment/mistrust factor. The SSP is relatively short compared with other similar instruments. Participants give answers on a 4-point Likert scale, ranging from 1 (not true at all) to 4 (exactly right).

The detachment/mistrust factor consists of the two subscales with the same names. These two subscales were selected for this study for measurement of convergent validity, based on the assumption that they would show a strong negative correlation with feelings of social safeness and pleasure. On the other hand, two subscales impulsiveness and adventure-seeking, used in the investigation of divergent validity, were assumed to show no or only small correlation with feelings of social safeness and pleasure. Cronbach’s alphas for the subscales detachment, mistrust, impulsiveness and adventure-seeking in the original validation study were 0.77, 0.78, 0.73, and 0.74, respectively [19]. The scales contain items such as “I feel best when I keep people at a certain distance” (detachment), “I tend to be on my guard with people who are somewhat more friendly than I expected” (mistrust), “I have a tendency to act on the spur of the moment without really thinking ahead” (impulsiveness) and “I have an unusually great need for change” (adventure-seeking). The SSP has shown good concurrent validity in relation to the five-factor model of personality [20].

#### The Hopkins Symptom Checklist (HSCL-25)

The Hopkins Symptom Checklist-25 (HSCL-25) is a shortened version of the original SCL-90, a self-report questionnaire designed to assess anxiety and depression. It consists of 25 items, divided into two subscales, with 10 items assessing symptoms of anxiety and 15 items assessing symptoms of depression. Participants indicate their answers on a 4-point Likert scale, ranging from 1 (not at all) to 4 (extremely). The scale has shown satisfactory validity and reliability [21] and Netteblad et al. [22] suggest a clinical a cut-off of 1.75, based on a Swedish study population. In the present study, the HSCL-25 was included to study levels of psychiatric symptoms in all three samples.

### Statistical analysis

Basic statistics such as analyzing normal distribution, skewness and kurtosis, were performed. Analyses showed that SSPS was normally distributed and showed only minor skewness and kurtosis, therefore, parametric tests were used.

A confirmatory factor analysis (CFA) was conducted to validate the previously identified one-factor solution in the original work [6]. As the exploratory factor analysis by Gilbert and colleagues were performed on a NC sample, we chose our NC sample for the main analyses of the CFA. Secondary analyses of the clinical groups were however also performed to further test the one-factor model.

Internal consistency was calculated with Cronbach’s alpha, with a recommended value of ≥ 0.70 [23]. Test–retest reliability was examined in the NC group with paired Pearson’s correlation coefficients, comparing ratings that were performed two weeks apart. The results were interpreted in accordance with Cohen’s recommendations, with all correlations above 0.50 considered strong [24].

Convergent and divergent validity was assessed by calculating Pearson’s correlation between the SSPS and the SSP subscales detachment and mistrust. The strength of the relationships between variables was evaluated based on Cohen’s criteria: small ≥ 0.10, medium ≥ 0.30 and large ≥ 0.50 [24].

Construct validity was explored through a comparison of the SSPS mean values by item in the NC sample and the clinical samples. Patients with ED and BPD were selected for the study because research has shown that low social safeness may be a common problem for people who experience these disorders. Analysis of variance was used to determine if there were statistical differences, and to assess differences in clinical symptomatology between the study populations. Complementary analyses were performed by also including the potential confounders gender, age, marital status, highest level of education and occupation in the model.

Analyses were performed using IBM SPSS Statistics, version 26.0. A significance level of 5% was used in all analyses.

## Results

### Factor structure and reliability

Data from the NC sample were used in the main analysis of the confirmatory factor analysis. Fit indexes suggested an acceptable fit between the model and the data: X^2^ (44, n = 407) = 160.193, *p* < 0.05, X^2^/df = 3.64, RMSEA = 0.081 (90% CI 0.067–0.094), SRMR = 0.041, CFI = 0.913. All items showed loadings between 0.72 and 0.78, except item four which had a factor loading of 0.47 (see Table 2). When running the CFA on the clinical sample, results were similar, however with an even better fit between the model and the data.

**Table 2:** Confirmatory factor analysis of the Swedish version of the Social Safeness and Pleasure Scale (SSPS)

Item no	Factor loading (Swe)	Factor loading (Eng, from Gilbert et al. 2009)	Item wording
1	0.74	0.51	I feel content within my relationships
2	0.78	0.63	I feel easily soothed by those around me
3	0.81	0.69	I feel connected to others
4	0.47	0.53	I feel part of something greater than myself
5	0.76	0.77	I have a sense of being cared about in the world
6	0.87	0.83	I feel secure and wanted
7	0.88	0.84	I feel a sense of belonging
8	0.77	0.80	I feel accepted by people
9	0.77	0.76	I feel understood by people
10	0.82	0.77	I feel a sense of warmth in my relationships with people
11	0.72	0.65	I find it easy to be calmed by people close to me

Analyses were performed in the non-clinical sample (n = 407). The factor loadings from the English original are provided for comparison purposes

Internal consistency was 0.95 in the whole sample, 0.94 in the NC and ED samples, and 0.88 in the BPD sample. Test–retest reliability was strong, with a Cronbach’s alpha of 0.92 in the NC sample.

### Validity

In the NC sample, SSPS was significantly and negatively correlated with the two SSP scales detachment (r = − 0.48, *p* < 0.001) and mistrust (r = − 0.53, *p* < 0.001) with moderate to strong correlations, supporting convergent validity (i.e., indicating that the scale correlates to a related construct). No correlation was found between SSPS and impulsivity (r = − 0.02, *p* = 0.67) and only a small correlation was found between SSPS and adventure-seeking (r = − 0.12, *p* < 0.05), supporting divergent validity (i.e., indicating that the scale does not correlate strongly to a distinct construct).

The comparison between the NC sample and the two clinical samples showed that the NC sample had lower levels of anxiety and depression than either clinical sample. However, the mean value of anxiety and depression in the NC sample was above the clinical cut-off of 1.75, suggesting that this sample was less representative of a NC population than the previous Swedish study population [22]. Further, the BPD sample had significantly higher levels of anxiety and depression symptoms than the ED sample (see Table 3).

**Table 3:** Comparison of SSPS and HSCL-25 mean values in the NC, ED, and BPD samples

Item	Mean (SD), NC sample (n = 407)	Mean (SD), ED sample (n = 103)	Mean (SD), BPD sample (n = 58)	F value	Tukey post hoc
SSPS	40.7 (9.6)	34.5 (10.4)	25.7 (8.0)	71.5	BPD*** < ED*** < NC***
HSCL-25	1.88 (0.63)	2.50 (0.63)	2.76 (0.49)	82.4	NC < ED***, NC < BPD***, ED < BPD*

*SSPS* Social Safeness and Pleasure Scale, *HSCL-25* The hopkins symptom checklist-25, *NC* Non-clinical, *ED* Eating disorder, *BPD* Borderline personality disorder

^*^ < 0.05, ** < 0.01, *** < 0.001

**Fig. 1:**
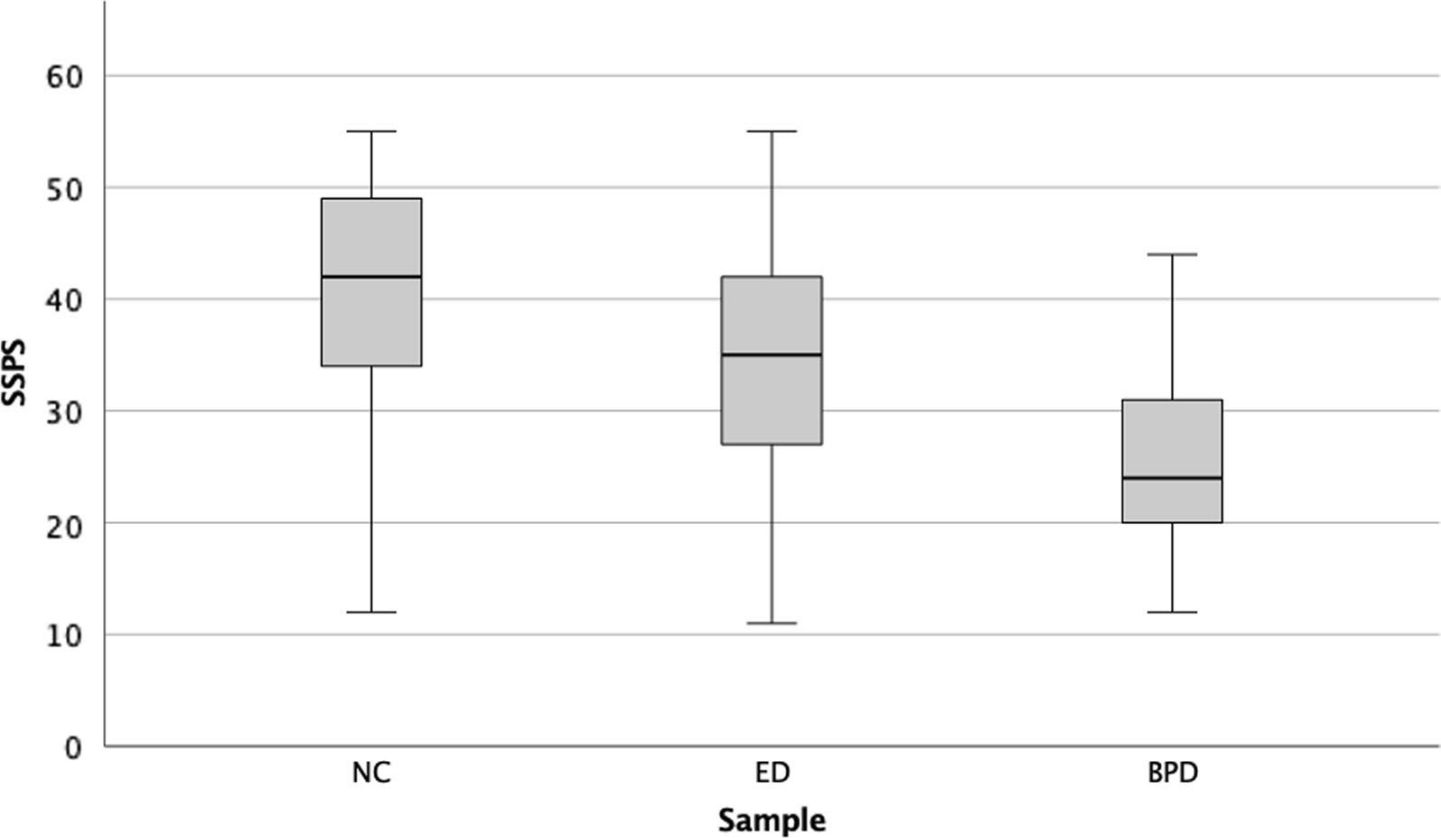
Boxplot of the Social Safeness and Pleasure Scale (SSPS) scores in the non-clinical (NC), eating disorder (ED), and borderline personality disorder (BPD) samples, respectively

When comparing the mean value of the SSPS in the NC sample with those in the clinical samples, the NC sample had significantly higher values for social safeness and pleasure than either the ED or the BPD sample, with the lowest values for social safeness and pleasure scored by the BPD sample, supporting construct validity (see Table 3 and Fig. 1). When including potential confounders in the model (i.e., demographic characteristics) the differences remained stable. Additional analyses were performed to see if results differed when the ED sample was separated into subgroups of AN, BN, and other EDs. Differences among ED groups were small and not significant.

## Discussion

The experience of being excluded from an important group that we want or need to belong to—a situation that has often been life-threatening during our evolutionary history—triggers strong aversive feelings in humans. It is therefore not surprising that we strive for a world that is safe, warm, and soothing, a world in which we experience social safeness and feel connected to others. The aim of the present study was to evaluate the psychometric properties of the Swedish version of the SSPS, a scale assessing the extent to which people experience their world as safe, warm, and soothing [6]. The main findings were that the Swedish version showed good to excellent psychometric properties. A factor analysis confirmed a one-factor structure and the scale showed high internal consistency, strong test–retest reliability, and satisfactory convergent and divergent validity. Also, social safeness was higher in a NC sample than in two clinical samples, while BPD patients reported lower levels of social safeness than ED patients [25].

The evaluation of the Swedish SSPS showed that internal consistency was high. This was in line with the evaluation of the original English version [6], as well as with evaluations of the SSPS in other languages [11]. The confirmation of a one-factor structure had support However, one item (no 4: ‘I feel part of something greater than myself’) was at the bottom end of the factor loading indicating a lesser contribution to the overall scale and a weaker association with the construct measured. This is in concordance with the original SSPS scale and could be reconsidered in a revision of the scale [6]. The test–retest correlation was strong, as expected, since social safeness has been suggested to be a stable and trait-like variable [7].

Convergent validity was confirmed by moderate to strong negative correlations between the individual items of the SSPS and the subscales detachment and mistrust in the SSP. Also, there were no or small correlations between the SSPS and the subscales impulsiveness and adventure-seeking, demonstrating divergent validity. The underlying assumption is that the detachment and mistrust subscales are in opposition to the construct of social safeness, whereas the subscales impulsiveness and adventure-seeking are not related to social safeness [19].

In previous studies, social safeness has been found to be distinguishable from both PA and NA, supporting discriminant validity [7], and difficulties in the development of social safeness have been suggested to be a transdiagnostic vulnerability factor [6, 12, 13]. Indeed, our results showed significantly lower scores for social safeness in our clinical samples compared with the NC sample. A possible conclusion from this is the importance for clinicians to focus on developing social safeness and other PA, in addition to their focus on reducing NA. This may be true in particular for patients with BPD, who show more severe difficulties in developing social safeness than ED patients. It may also be important to support patients in their interactions with supportive others on a regular basis, since this is believed to increase the level of social safeness [7]. Another reason to focus more on social safeness and other PA in psychotherapeutic treatments is that some may be fearful of experiencing PA which may lead to experiential avoidance. Focusing too much on reducing NA and not on increasing PA can prevent successful therapy. For example, fear of happiness was found to be a strong predictor of depression, anxiety, and stress in a depressed sample, with levels significantly higher than in a group of students [2].

### Implications and suggestions for future research

Difficulties in experiencing social safeness have been reported to correlate with psychiatric problems and are believed to be a transdiagnostic vulnerability factor. In the long term, the lack of connectedness that this leads to may increase the risk of loneliness [26]. Further, social safeness has been related to difficulties in emotion regulation. More specifically, social safeness and pleasure has been suggested to be some kind of buffer against maladaptive mood dysregulation [6], i.e., the higher social safeness and pleasure, the less the individuals are affected by their mood disorder. Difficulties in emotion regulation are associated with a range of psychiatric disorders and mental health problems, particularly depression and bipolar disorder [1, 6, 27, 28]. There is also broad evidence for emotion regulation difficulties in individuals diagnosed with borderline personality disorder (BPD), anorexia nervosa (AN), bulimia nervosa (BN), substance abuse, attention-deficit hyperactivity disorder, and autism spectrum disorder [29, 30]. If increased social safeness and pleasure was included as an important aspect of recovery, difficulties in emotion regulation may have less impact on these individuals. Moreover, items of the SSPS are highly overlapping with the intimacy domain of the Alternative DSM-5 Model for Personality Disorders [31], further highlighting the importance of good social relationships for the sense of well-being. The SSPS is a valid and reliable instrument that can be used in both clinical practice and research to investigate the concept of social safeness and pleasure. For example, it could play an important role in the evaluation of therapeutic interventions that target the ability to develop social safeness. Examples include compassion-focused therapy; helping patients decrease self-criticism and become more compassionate [2], and radically open dialectical behavior therapy; helping patients become more flexible and open-minded, show pro-social signals, and form close social bonds [26, 32, 33]. The SSPS could also lead to new insights regarding how to downregulate the threat system in a range of mental disorders.

### Limitations

The study has several limitations. First, the differences between the NC and clinical samples with regard to social safeness might be greater than has been reported, since the NC sample showed a level of anxiety and depression above the clinical cut-off, i.e., it displayed more psychiatric problems than expected and compared with the general population in Sweden. The reason for this might be that the convenience sample potentially included many participants with particular interest in psychology and mental health, possibly because of personal experience in this area. Nevertheless, the study included two different clinical samples and a significant difference was seen between these two groups and the NC sample. Further, even though the three distinct samples are a strength, the somewhat small size of the BPD sample (n = 58) might be a limitation. Second, the study does not add any information on the questionnaire’s sensitivity to change. Third, there are limitations with self-rating scales. For example, people may want to give the impression of experiencing social safeness to a large extent, since a lack of connectedness and loneliness are often linked to feelings of shame. Adding a clinically rated instrument would have strengthened the findings. Fourth, it was not assessed if patients in the ED sample also suffered from BPD, or if patients in the BPD sample also suffered from ED. ED levels among those with BPD have been reported to be as high as 50% [34], complicating the interpretation of our findings. However, excluding these individuals would have made the group less representative of the actual clinical BPD group, and it is important to remember that BPD was their main diagnosis. Furthermore, the prevalence of BPD among those with ED is lower, ranging from 2 to 26% [35, 36]. Lastly, the clinical samples consisted mainly of women; therefore, the results should not be generalized to a population of men.

## Conclusions

Our findings support the continued use of the Swedish version of the SSPS as a reliable and valid instrument in the assessment of social safeness.

## Data Availability

Data will not be made publicly available due to confidentiality, but can be made available upon reasonable request to the corresponding author.

## Abbreviations

AN: Anorexia nervosa
BN: Bulimia nervosa
BPD: Borderline personality disorder
ED: Eating disorder
HSCL-25: Hopkins symptom checklist
KMO: Kaiser–Meyer–Olkin
NA: Negative affect
NC: Non-clinical
PA: Positive affect
SCID-II: Structured clinical interview for DSM-IV axis II personality disorders
SSPS: Social Safeness and Pleasure Scale
SSP: Swedish Universities Scales of Personality
